# Early neonatal loss of inhibitory synaptic input to the spinal motor neurons confers spina bifida-like leg dysfunction in a chicken model

**DOI:** 10.1242/dmm.031054

**Published:** 2017-12-01

**Authors:** Md. Sakirul Islam Khan, Hiroaki Nabeka, Farzana Islam, Tetsuya Shimokawa, Shouichiro Saito, Xuan Li, Soichiro Kawabe, Fumihiko Hamada, Tetsuya Tachibana, Seiji Matsuda

**Affiliations:** 1 Department of Anatomy and Embryology, Graduate School of Medicine, Ehime University, Toon 791-0295, Ehime, Japan; 2 Department of Animal Science, Bangladesh Agricultural University, Mymensingh 2202, Bangladesh; 3 Laboratory of Veterinary Anatomy, Faculty of Applied Biological Sciences, Gifu University, Yanagido, Gifu 501-1128, Japan; 4 Fukui Prefectural Dinosaur Museum, Katsuyama, Fukui 911-8601, Japan; 5 Department of Human Anatomy, Faculty of Medicine, Oita University, Yufu, Oita 879-5593, Japan; 6 Department of Agrobiological Science, Faculty of Agriculture, Ehime University, Matsuyama 790-8566, Japan

**Keywords:** Neural tube defect, Chicken model, Synaptic inputs, GABAergic transmission, Neurodegeneration, Motor dysfunction

## Abstract

Spina bifida aperta (SBA), one of the most common congenital malformations, causes lifelong neurological complications, particularly in terms of motor dysfunction. Fetuses with SBA exhibit voluntary leg movements *in utero* and during early neonatal life, but these disappear within the first few weeks after birth. However, the pathophysiological sequence underlying such motor dysfunction remains unclear. Additionally, because important insights have yet to be obtained from human cases, an appropriate animal model is essential. Here, we investigated the neuropathological mechanisms of progression of SBA-like motor dysfunctions in a neural tube surgery-induced chicken model of SBA at different pathogenesis points ranging from embryonic to posthatch ages. We found that chicks with SBA-like features lose voluntary leg movements and subsequently exhibit lower-limb paralysis within the first 2 weeks after hatching, coinciding with the synaptic change-induced disruption of spinal motor networks at the site of the SBA lesion in the lumbosacral region. Such synaptic changes reduced the ratio of inhibitory-to-excitatory inputs to motor neurons and were associated with a drastic loss of γ-aminobutyric acid (GABA)ergic inputs and upregulation of the cholinergic activities of motor neurons. Furthermore, most of the neurons in ventral horns, which appeared to be suffering from excitotoxicity during the early postnatal days, underwent apoptosis. However, the triggers of cellular abnormalization and neurodegenerative signaling were evident in the middle- to late-gestational stages, probably attributable to the amniotic fluid-induced *in ovo* milieu. In conclusion, we found that early neonatal loss of neurons in the ventral horn of exposed spinal cord affords novel insights into the pathophysiology of SBA-like leg dysfunction.

## Abstract (precis)

**Summary:** Consistent with the symptoms of humans with SBA, chickens with SBA-like features exhibit lower-limb paralysis within two weeks after hatching, which appeared to coincide with synaptic change-induced disruption of spinal motor networks.

## INTRODUCTION

Spina bifida aperta (SBA), also known as myelomeningocele, is one of the most common congenital malformations, causing lifelong disability. SBA develops in ∼1/1000 neonates worldwide; the lifetime cost is more than US$600,000 (Copp et al., 2015). SBA is characterized primarily by defective fusion of the neural tube, which causes *in utero* damage to the exposed spinal cord (Osaka et al., 1978; Copp et al., 1990; Heffez et al., 1990; Millicovsky and Lazar, 1995). Fetuses with SBA exhibit voluntary leg movements *in utero* and during early neonatal life, but these disappear within the first few weeks after birth (Korenromp et al., 1986; Sival et al., 1997, 2004, 2006, 2008). *In utero* surgical closure of the exposed cord seeks to preserve spinal tissue and improve motor dysfunction (Meuli et al., 1995a, 1996; Sival et al., 1997; Adzick et al., 1998; Tulipan et al., 1999); the procedure reduces some SBA-related neurological complications but does not preserve motor function in humans (Tulipan et al., 1999; Sival et al., 2004; Fichter et al., 2008; Adzick, 2013). The direct causes of such dysfunction remains unclear; ethical and technical limitations render it difficult to obtain information from humans. Thus, research in analogous animal models of SBA is needed to better understand the cellular and molecular mechanisms of leg dysfunction and to develop novel therapeutic interventions.

**Fig. 1.**
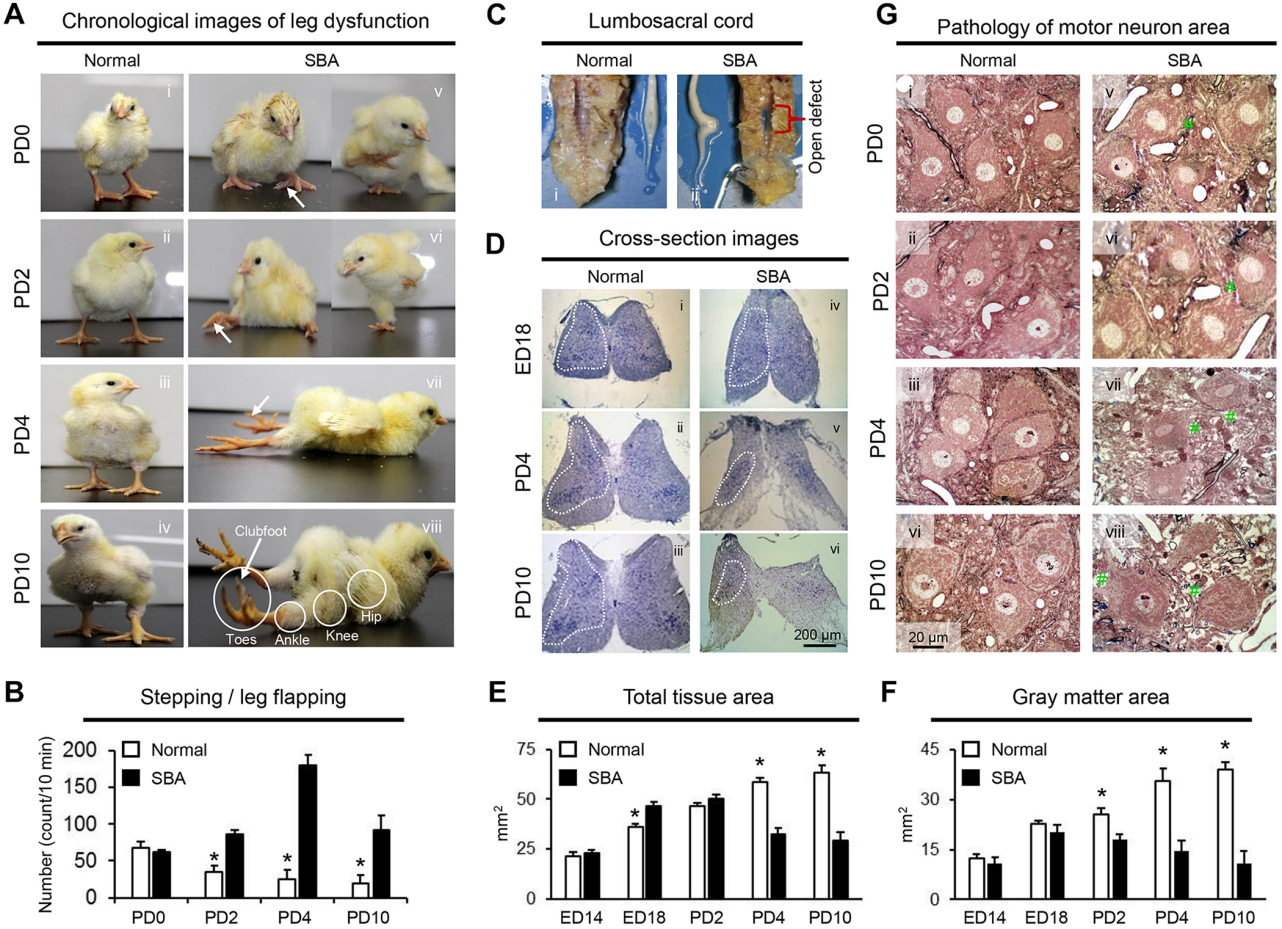
**Assessments of motor behavior and spinal cord gross histology at the lesion sites in SBA chicks.** (A) Representative photographs showing the phenotypes of normal (i-iv) and SBA (v-viii) chicks. The normal control chicks were able to stand firmly shortly after hatching (i). The SBA chicks were able to put their toes on a surface and had the ability to sit-to-walk at PD0 (v), but this gradually deteriorated with age (days), until the chicks completely lost the functional ability to sit-to-walk from PD4 (vii,viii). Severe clubfoot deformities (arrows) appeared in both legs of SBA chicks within 10 days of hatching. (B) Total numbers of leg movements (stepping and/or leg flapping/hitching) per 10 min in normal and SBA chicks. (C) Gross necropsy revealed open wounds (SBA lesion) on the lumbosacral region of the back of the SBA chicks as well as varying degrees of spinal cord deformation in the lesion area (ii, location of open defect). (D) Cross-sectional images of exposed cord from SBA chicks stained with Hematoxylin-Eosin, showing varying degrees of alterations in anatomical shape (iv-vi); this was not observed in the normal chicks (i-iii). (E,F) Total tissue area and gray matter area in the exposed lumbar cord (location of open defect) of SBA chicks and in a similar location in normal chicks on the embryonic and neonatal days. (G) Pathological alterations in the lumbar cord motor neuron area of SBA chicks. Representative photographs of semi-thin Toluidine-Blue-stained sections from the exposed lumbar cord of SBA chicks (iv-vi) and in a similar location in normal control chicks (i-iii) on neonatal days. Vacuolated and swollen axons and feasible dendrites (hash signs) were evident in SBA chicks on posthatch days. The data in B, E and F are presented as mean±s.e.m.; *n*=6 in each group at each age point. *P<0.01, in comparison to the SBA group at each age point; two-way ANOVA and post hoc Tukey's test.

Surgically induced exposure of the spinal cord causes human-like spina bifida lesions in different mammals (rat, pig and sheep) triggering *in utero* damage to the exposed spinal cord and various neurological disorders (Heffez et al., 1993; Meuli et al., 1995a,b). A criticism of such models is that laminectomy is performed from middle to advanced gestation, limiting the experimental relevance to secondary spinal cord injuries induced by amniotic fluid in early gestation. Thus, studies in these animal models have focused principally on optimizing surgical coverage, refining fetal surgery techniques and evaluating tissue-engineering approaches (Meuli and Moehrlen, 2014; Papanna et al., 2016; Watanabe et al., 2016). Several genetic (Sim et al., 2013; Nikolopoulou et al., 2017) and drug-induced (Alles and Sulik, 1990; Danzer et al., 2005) rodent models of neural tube-related disorders exist, but are of limited utility in SBA research on postnatal leg dysfunction because the fetuses generally die *in utero* (Copp et al., 1982; Selçuki et al., 2001). However, incision of the roof plate of the chicken neural tube during early gestation triggers *in ovo* exposure of the spinal cord *ab initio* (Mominoki et al., 2006; Tsujimura et al., 2011). Also, SBA-like features including motor dysfunction, quite similar to those of human neonates, are evident during neonatal life (Fig. 1, Table 1). Thus, the chicken model should yield valuable data on the pathological sequence of events in the spinal cord associated with SBA-like motor dysfunction.

Many neurological complications, including motor dysfunction, at postnatal age have been associated with alterations of synaptic transmission in spinal motor networks, mainly dysregulation of the balance between excitation and inhibition (Schütz, 2005; Sunico et al., 2011; Ramírez-Jarquín et al., 2014). It is postulated that alterations in synaptic transmission, particularly inhibitory neurons, by spinal motor neurons could be associated with SBA-related motor dysfunction because changes in the extent of γ-aminobutyric acid (GABA)ergic activities, the principal inhibitory modulator, play key roles in stepping (Sinnamon and Benaur, 1997), hind limb paralysis (Gottesfeld et al., 1976) and motor dysfunction (Sanna et al., 1993; Ramírez-Jarquín et al., 2014). Thus, we explored the excitatory and inhibitory synaptic inputs to spinal cord motor neurons at different ages ranging from gestation to posthatching in a chicken SBA model.

## RESULTS

### Motor behavior and neurological assessments

Neurophysiological dysfunction was evident from the day of hatching [postnatal day (PD) 0] (Fig. 1A, Table 1). Shortly after hatching, the chicks with SBA could stand, move the leg joints and walk, but the voluntary control of movement (sitting to walking) was poor compared with that of normal chicks. Over the next 2 weeks, the SBA chicks lost function in the toe, ankle and knee joints, and subsequently developed lower-limb paralysis (Fig. 1A, Table 1), although concurrent leg flapping and/or hitching, principally in the hip joints, was evident during wing, head and neck movements (Fig. 1B). In addition, severe clubfoot deformities appeared in both legs within the first 2 weeks of neonatal life (Fig. 1A, arrows).

**Table 1.**
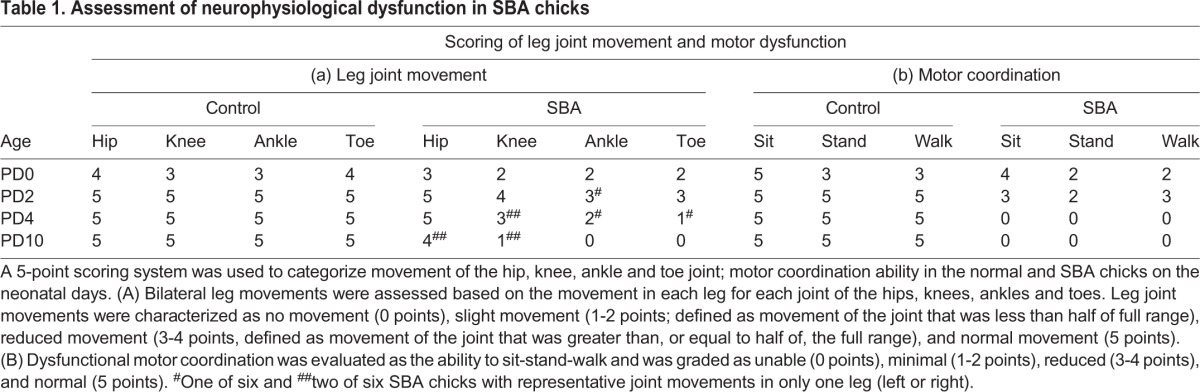
**Assessment of neurophysiological dysfunction in SBA chicks**

### Histopathological analysis

To explore whether spinal cord exposure to amniotic fluid triggered progressive destruction of neural tissue, we compared cord sections at the SBA lesion to those of normal controls at times ranging from embryonic day (ED) 14 to PD10. Gross necropsy of SBA chicks revealed open wounds (SBA-like lesions) in the lumbosacral region of the back, varying degrees of spinal cord deformation in the lesional area (Fig. 1C), quite similar to human myelomeningocele (Hutchins et al., 1996). The area of greatest compression (the gross lesion epicenter) was at L2-L4 (the open defect) (Fig. 1C,ii). Apart from the changes in anatomical shape, cross-sectional images of the lesional epicenter revealed prominent tissue loss, particularly the dorsal horn (Fig. 1D-F). The extent of deformation of the neural tissue area (gray matter) in the exposed spinal cord varied by age, being similar during the embryonic period, then increasing with age after hatching and becoming severe at PD10. Furthermore, the number of motor neurons seemed to be preserved during the embryonic period and the early neonatal days (ED14 to PD4) but had decreased by PD10 (Fig. 5B). Another prominent change was large vacuolation between motor neurons at PD10. These changes were not noted earlier in SBA chicks or in control chicks of any age (Fig. 1G), and led to the loss of motor neuron synaptic terminals (Sunico et al., 2011).

### Excitatory and inhibitory synaptic boutons on spinal cord motor neurons

**Fig. 2.**
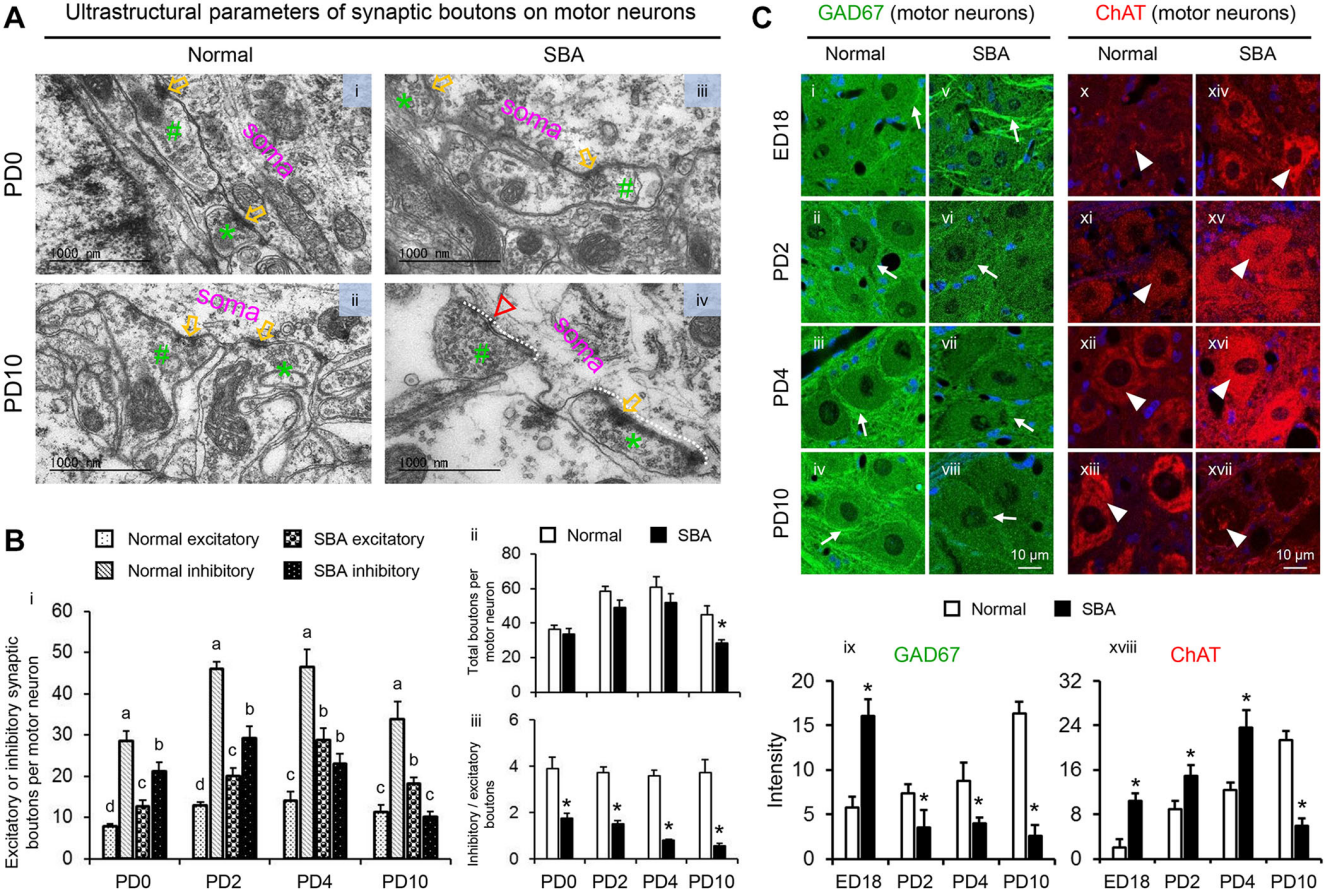
**Postnatal loss of inhibitory synaptic transmission in the spinal cord motor neurons of SBA chicks.** (A) Illustrative examples of synaptic boutons attached to the plasma membrane of motor neurons with either spherical (asterisks, excitatory) or flat/pleomorphic (hash signs, inhibitory) vesicles. Dotted lines indicate the segments of the bouton that were detached from the motor neuron cell body; arrows indicate the active zones; triangle indicates the absence of active zones. (B) Characterized by the type of vesicles (i, excitatory or inhibitory boutons), average number of synaptic boutons (ii) and ratio of inhibitory-to-excitatory synaptic boutons per motor neuron (iii) from chicks with the indicated genotypes at the indicated conditions. The data in B are presented as mean±s.e.m.; 10 motor neurons from each chick were analyzed at each age point; there were three chicks at each age point in each group. (C) GABA synthesis, glutamic acid decarboxylase 67 (GAD67; i-viii, arrows), and acetylcholine synthesis, choline acetyltransferase (ChAT; x-xvii, arrowheads) immunoreactivities in the lumbar cords of normal and SBA chicks at different developmental stages. Representative confocal images from the ventral horn of the open defect in the lumbar cord of SBA chicks and in a similar location in normal controls at ED18, PD2, PD4 and PD10. Each panel represents the immunoreactivities of GAD67 (i-viii, green) and DAPI (blue) or ChAT (x-xvii, red) and DAPI (blue). The staining intensities of GAD67 and ChAT in the ventral horn (motor neuron area) of the open defect lumbar cord in SBA chicks, and in a similar location in normal chicks, at different age points were measured using ImageJ software. The intensity data in C are presented as mean±s.e.m. Six random sections per chick at each age point in each group were analyzed for quantification; there were six chicks in each group at each age point. *P<0.05, in comparison to the normal control group at each age point (B,ii,iii; C,ix,xviii). Different letters indicate significant differences between groups at each age point (B,i); two-way ANOVA, post hoc Tukey's test.

To clarify whether actual synaptic alterations took place on the lumbar cord motor neurons in SBA chicks during early neonatal days, we used electron microscopy to analyze the ultrastructural parameters of synaptic boutons attached to motor neurons of the exposed lumbar cord (Fig. 2A). In control chicks, segments of the synaptic boutons were attached to the motor neuron cell body, but the boutons were detached in SBA chicks at severe symptomatic stage on PD10. On PD10, active zones were observed in excitatory synaptic boutons of SBA chicks but not in inhibitory boutons, indicating loss of inhibitory inputs on motor neurons (Sunico et al., 2011).

Quantitative analyses revealed that the average number of synaptic boutons per motor neuron in SBA chicks did not differ from that of controls on PD0, PD2 or PD4 (Fig. 2B,ii). However, SBA chicks had significantly greater numbers of excitatory boutons and fewer inhibitory boutons than age-matched controls, commencing from the day of hatching (Fig. 2B,i). The proportions of inhibitory and excitatory boutons on motor neurons of normal neonatal chicks were ∼75% and 25%, respectively; these proportions were not evident in SBA chicks at any time. In fact, the value of inhibitory-to-excitatory boutons per motor neuron was significantly lower in SBA chicks than in age-matched normal controls from PD0, and gradually decreased with progression of leg dysfunction; this was drastic on PD10 (Fig. 2B,iii), when SBA chicks completely lost motor coordination and their voluntary leg movements and showed lower-limb paralysis (Fig. 1A,B, Table 1).

### Excitatory and inhibitory immunoreactivities of spinal cord motor neurons

To explore whether changes in the inhibitory and excitatory inputs to motor neurons were associated with the levels of excitatory and inhibitory neurotransmitters, and whether such changes commenced during embryogenesis or after hatching, we conducted immunostaining analyses using markers of excitatory [acetylcholine synthesis, choline acetyltransferase (ChAT)] and inhibitory [GABA synthesis, glutamic acid decarboxylase 67 (GAD67)] transmission, at different time points from ED18 to PD10 (Fig. 2C). In normal chicks, both immunoreactivities were low in advanced gestation but increased after hatching (Fig. 2C,i-iv,x-xiii). However, clear increases in immunoreactivities at the open defect spinal cord of SBA chicks were evident at ED18 (Fig. 2C,v, xiv), indicating that the synaptic abnormalities commenced during embryonic development. Furthermore, in line with the electron microscopy data (Fig. 2B,i), ChAT immunoreactivity increased, whereas that of GAD67 decreased, on PD2 and PD4 (Fig. 2C,vi,vii,xv,xvi), but both had decreased by PD10, at which time GAD67 immunoreactivity was almost absent (Fig. 2C,viii,xvii). When we compared immunoreactivity intensity, a significantly higher level of ChAT was observed in motor neurons of SBA chicks than in age-matched normal controls during early neonatal days (Fig. 2C,xviii). On the other hand, the GAD67 expression level around motor neurons of SBA chicks was significantly decreased during posthatch days (Fig. 2C,ix), suggesting an association between GABAergic transmission and reduction of inhibitory inputs to motor neurons.

### GABAergic transmission by spinal cord motor neurons

**Fig. 3.**
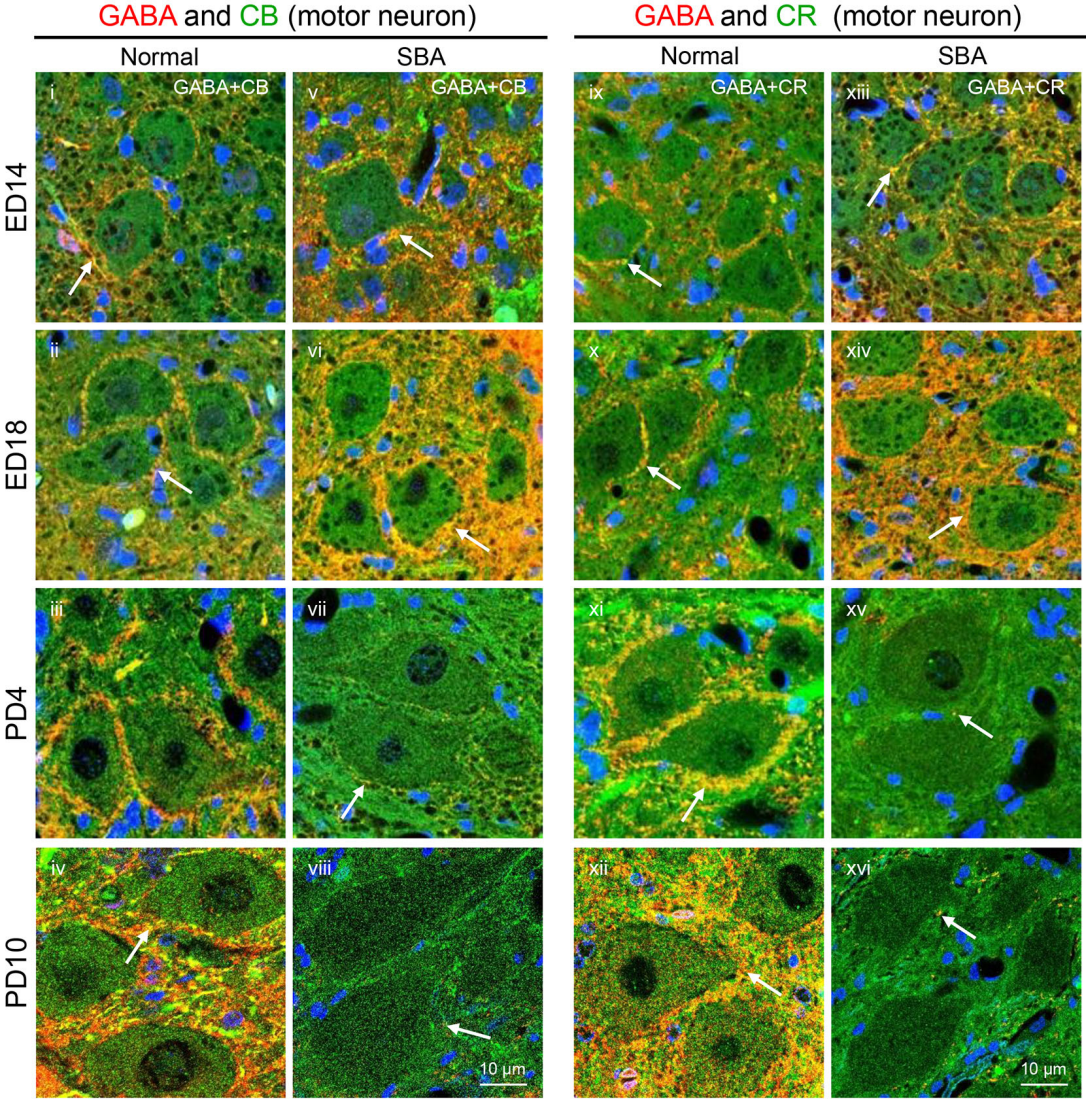
**Postnatal loss of GABAergic terminals around motor neurons in the exposed lumbar cord of SBA chicks.** (A) Double-labeled immunofluorescent staining performed with antibodies directed against GABA and either CB or CR. Representative confocal images showing the localization patterns of GABA and CB (i-viii, arrows) or GABA and CR (ix-xvi, arrows) in the normal and SBA chicks on ED14, ED18, PD4 and PD10. Images of the ventral horn in the open defect of the lumbar cord in SBA chicks and in a similar location in normal chicks. Each panel displays the immunoreactivities of GABA (red) and CB or CR (green), with sites of colocalization (yellow) and DAPI (blue).

To explore whether a reduction of inhibitory inputs to motor neurons was associated with the loss of GABAergic transmission, we immunohistochemically analyzed GABA and its subpopulations, calbindin-D-28K (CB) and calretinin (CR), at different time points (ED14 to PD10). For GAD67, more GABA-synaptic terminals were observed on exposed cord motor neurons at ED14 and ED18 in SBA chicks than in age-matched controls. These gradually declined with posthatch age and were almost absent at PD10 (Fig. S2,i-x). When we compared immunoreactivity intensity, a significantly higher level of GABA was observed around motor neurons in SBA chicks than in age-matched normal controls at ED18 (control, 11.46±1.21; SBA, 26.52±1.70; *P*<0.01, Student's *t*-test). This immunoreactivity gradually decreased with posthatch age and was drastic at PD10 (control, 30.59±1.18; SBA, 2.05±1.27; *P*<0.01, Student's *t*-test), suggesting that both GABA synthesis and release gradually decreased in exposed cords as motor dysfunction progressed. Similarly, the immunoreactivities of both CB (Fig. S2,xi-xx) and CR (Fig. S2,xxi-xxx) gradually fell after hatching. Furthermore, double labeling of GABA and either CB or CR was prominent during middle and late gestation, but was greatly reduced at PD4 and almost absent at PD10 (Fig. 3), at which time a drastic decrease in the number of inhibitory synaptic boutons on motor neurons was evident (Fig. 2B,i).

### Neurodegeneration in the spinal cord

**Fig. 4.**
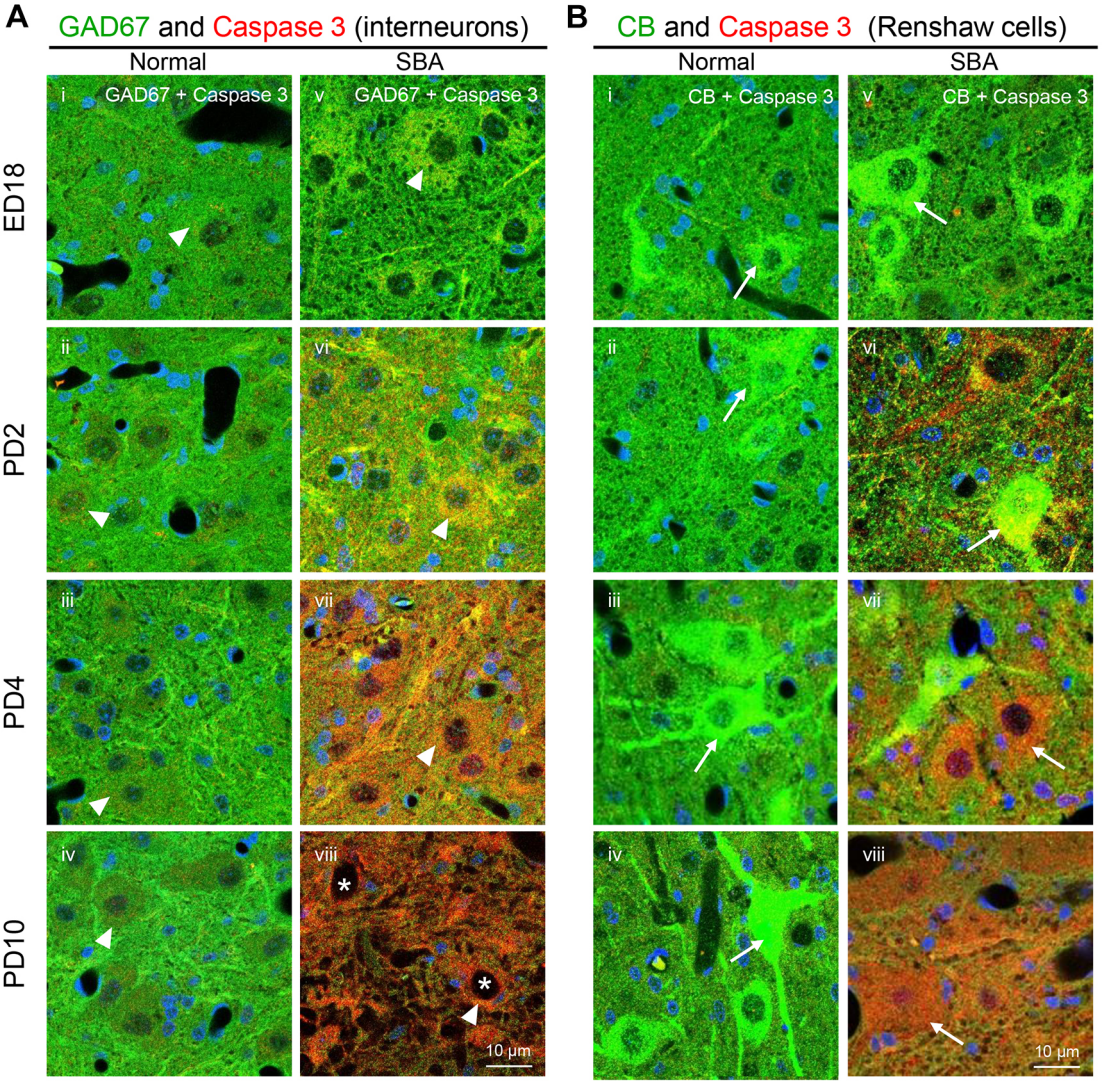
**Caspase 3-mediated apoptosis of spinal cord GABAergic interneurons in SBA chicks.** (A) Representative confocal images showing immunofluorescence staining of caspase 3, a marker of apoptosis, and GAD67, a GABA-synthesizing enzyme, in the ventral horn interneurons (arrowheads) from the location of the open defect in the lumbar cord of SBA chicks (v-viii) and in a similar location in normal chicks (i-iv). (B) Representative confocal images showing immunofluorescence staining of caspase 3 and CB-expressing Renshaw cells (arrows) in normal (i-iv) and SBA (v-viii) chicks. Images of the ventral horn in the open defect of the lumbar cord in SBA chicks and in a similar location in normal chicks. Each panel displays the immunoreactivities of caspase 3 (red) and GAD67 or CB (green), with sites of colocalization (yellow) and DAPI (blue). Asterisks indicate interneuron-like damaged structures observed in SBA chicks at PD10.

To clarify the mechanisms underlying the loss of GABAergic synaptic terminals on motor neurons in SBA chicks, the immunoreactivity of caspase 3, a marker of apoptosis, in interneurons expressing GAD67 and CB was evaluated in the ventral horn of the lumbar cord at different pathogenesis points (Fig. 4). We observed pathogenesis point-dependent changes in caspase 3 immunoreactivities in GABAergic interneurons in the SBA chicks, such that weak caspase 3 immunoreactivity was evident in GAD67-expressing interneurons at ED18 (Fig. 4A,v) while moderate to high caspase 3 immunoreactivity was found on PD2 and PD4 (Fig. 4A,vi,vii). However, most of these interneurons had likely degenerated by PD10 because only a few interneuron-like structures remained at this point (Fig. 4A,viii, asterisks). Similar patterns of caspase 3 expression were observed in CB-expressing Renshaw cells of SBA chicks (Fig. 4B,v-viii). In addition to the GABAergic interneurons, strong caspase 3 immunoreactivity was observed in the glial cells and tissue area of the exposed cord in SBA chicks on PD4 (Fig. 4A,vii), suggesting loss of glial cells and tissue area owing to apoptosis by PD10.

**Fig. 5.**
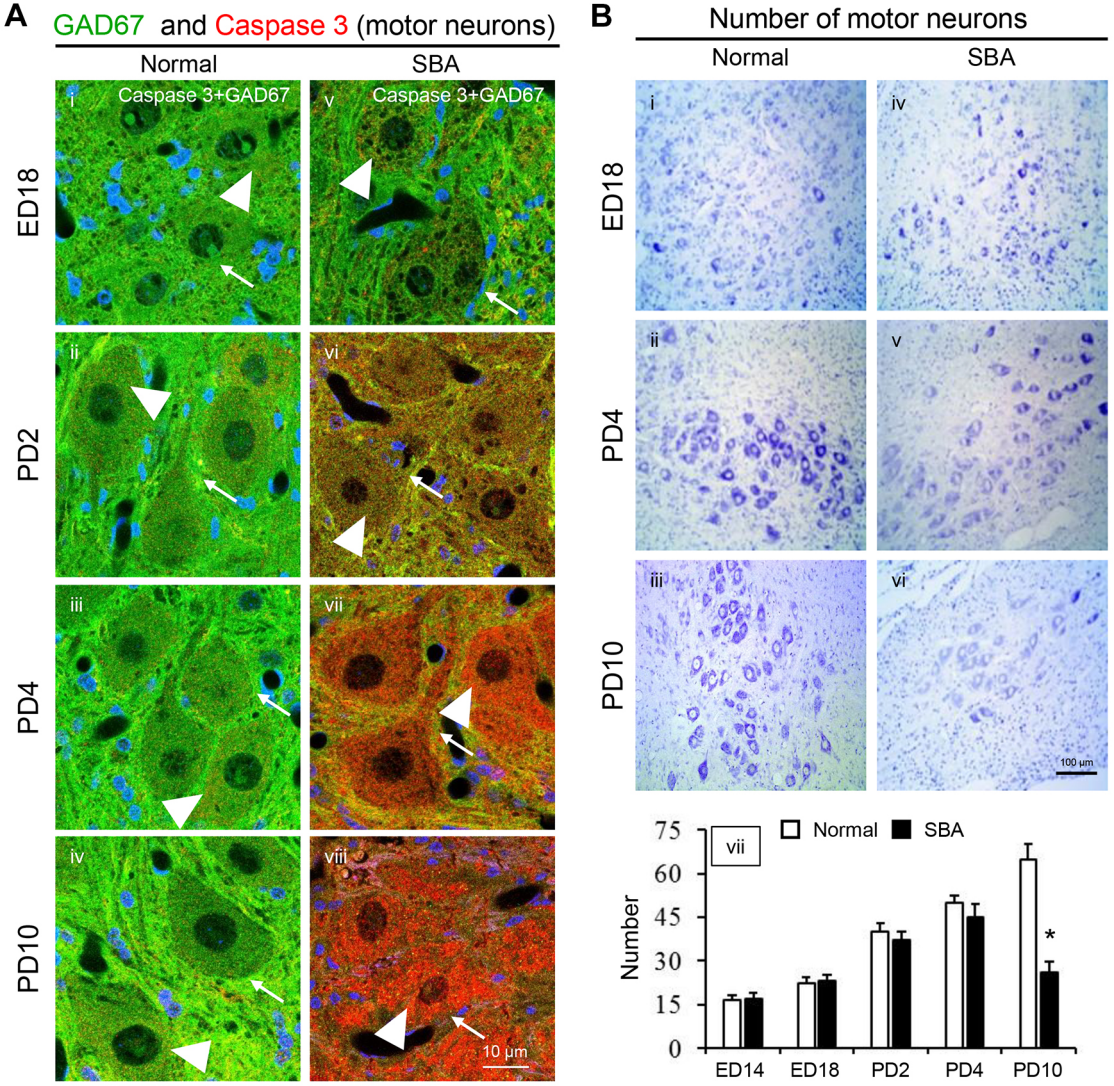
**Caspase 3-mediated apoptosis of** **spinal cord motor neurons in SBA chicks.** (A) Representative confocal images showing immunofluorescence staining of caspase 3 and GAD67. Images of the ventral horn in the open defect of the lumbar cord in SBA chicks (v-viii) and in a similar location in normal chicks (i-iv). Each panel displays the immunoreactivities of caspase 3 (red, arrowheads), GAD67 (green, arrows) and DAPI (blue). (B) Loss of motor neurons in the lumbar cord of SBA chicks. Nissl-stained images obtained from the ventral horn of the location of the open defect in the lumbar cord of SBA chicks (iv-vi), and in a similar location in normal chicks (i-iii), at different age points. Numbers of motor neurons in the ventral horn of the lumbar spinal cord sections of SBA and normal control chicks (vii). The numbers of motor neurons were calculated and averaged across six cross sections per chick at each age point; there were six chicks at each age point in each group. Data are expressed as mean±s.e.m. *P<0.01, in comparison to the normal control group; two-way ANOVA, post hoc Tukey's test.

We also analyzed the immunoreactivity of caspase 3 on motor neurons to determine whether apoptosis was associated with their degeneration in SBA chicks (Fig. 5). As in the GABAergic interneurons, weak caspase 3 immunoreactivity was observed in the motor neurons of SBA chicks at ED18 (Fig. 5A,v), which increased with posthatch age. From PD4, abundant immunoreactivity was evident, and was very strong on PD10 (Fig. 5A,vii,viii), which is when SBA chicks had only approximately half the number of motor neurons in exposed cords as age-matched normal controls (Fig. 5B,vii). On the other hand, very little immunoreactivity was identified in either GABAergic interneurons (Fig. 4A,i-iv and B,i-iv) or motor neurons (Figs. 5A,i-iv) of normal chicks during the experimental period, suggesting that the degeneration of GABAergic interneurons and motor neurons in the lumbar cord of SBA chicks might occur via apoptosis.

### GABAergic, cholinergic and caspase 3 activities in sham control chicks

**Fig. 6.**
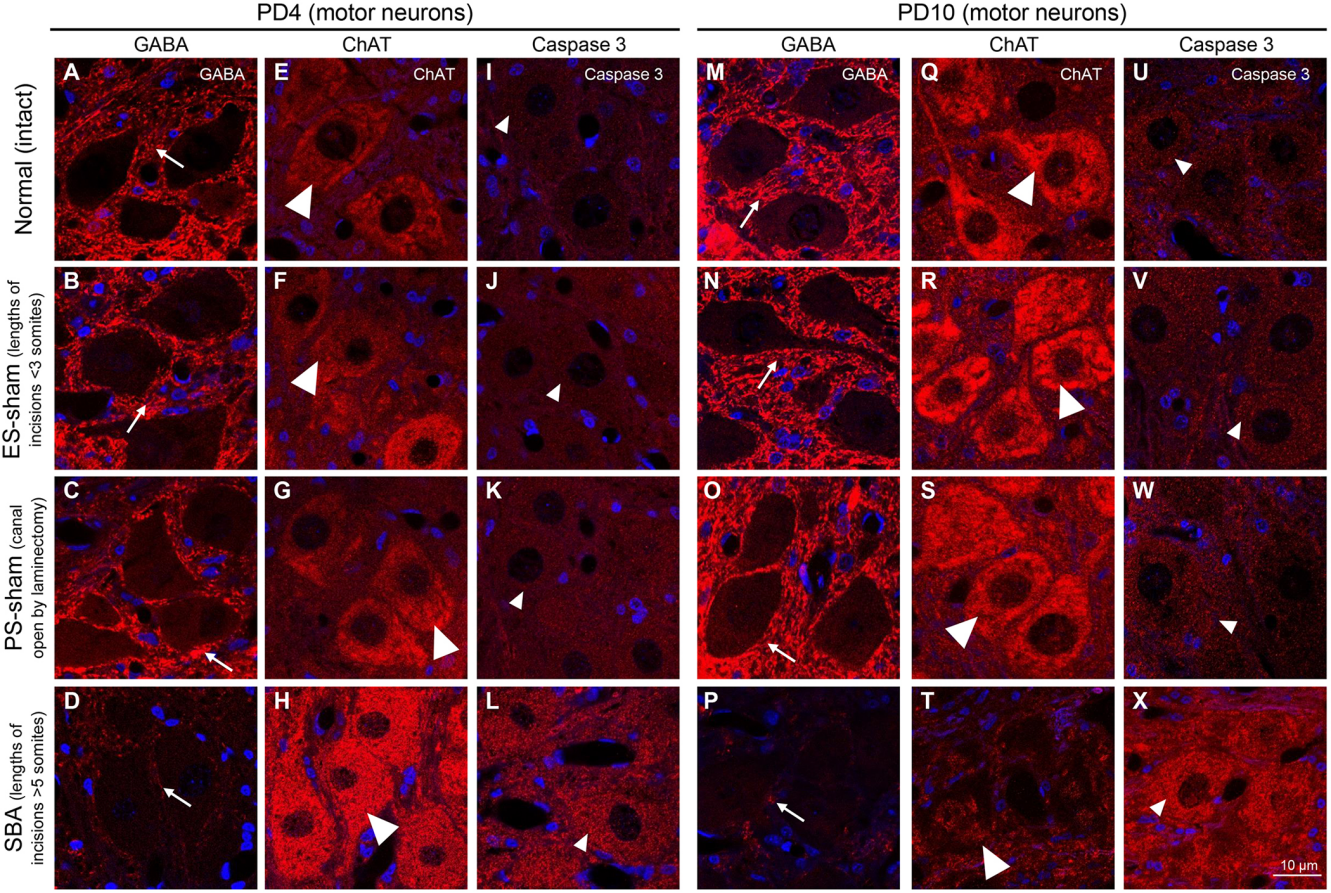
**Mechanical injuries at the embryonic and posthatch stages did not influence GABAergic, cholinergic or caspase 3 reactivity in the sham control chicks.** Representative confocal images from the ventral horn at the location of the open defect in the lumbar cord of SBA chicks and in similar locations in the normal controls, ES-sham and PS-sham chicks at PD4 and PD10. Each panel displays the immunoreactivities of GABA (a-d,m-p; red, arrows) and DAPI (blue) or ChAT (e-h,q-t; red, large arrowheads) and DAPI (blue), or caspase 3 (i-l,u-x; red, small arrowheads) and DAPI (blue).

To clarify whether mechanical and environmental insults played roles in the changes in GABAergic, cholinergic, and apoptosis activities, the immunoreactivities of GABA, ChAT and caspase 3 were compared between SBA chicks and two sham-injured groups: the ES-sham group, in which embryos received the same treatment as the SBA group except that the length of the incision of the roof plate was less than three somites; and the PS-sham group, in which an SBA-like open defect in the back of the chicks was made on PD0 via laminectomy at three vertebral segments, L2-L4 (Fig. S1B,C). Although PS-sham chicks showed reduced ability to walk during the first 2 days after laminectomy, their voluntary leg movements on PD10 remained functional. Neither sham group showed spinal cord deformation or changes in tissue area at the lesion sites. In addition, sham injuries did not alter the expression levels of GABA, ChAT or caspase 3 in the lumbar cord motor neurons on E14, E18, PD4 or PD10 (Fig. 6; Fig. S4). These findings suggest that the mechanical and environmental insults had little or no influence on GABAergic, cholinergic and caspase 3 activities in SBA chicks; rather, the changes of synaptic transmission and neurodegeneration were likely a consequence of secondary and/or tertiary effects of spinal cord exposure to amniotic fluid during *in ovo* development (Meuli et al., 1995a; Hutchins et al., 1996; Stiefel et al., 2007; Adzick, 2013; Kowitzke et al., 2016).

## DISCUSSION

Chicks with SBA-like features initially had normal voluntary leg movements but quickly after hatching (within 2 weeks) exhibited lower-limb paralysis. Such motor dysfunction has also been observed in human neonates with SBA (Korenromp et al., 1986; Sival et al., 2004, 2006, 2008). However, why these changes occur within this short period of time remains to be determined. Because there are no genetic or drug-induced factors involved in the exposure of the spinal cord to amniotic fluid, our results could be relevant for understanding the pathological events responsible for the progression of SBA-like leg dysfunctions.

**Fig. 7.**
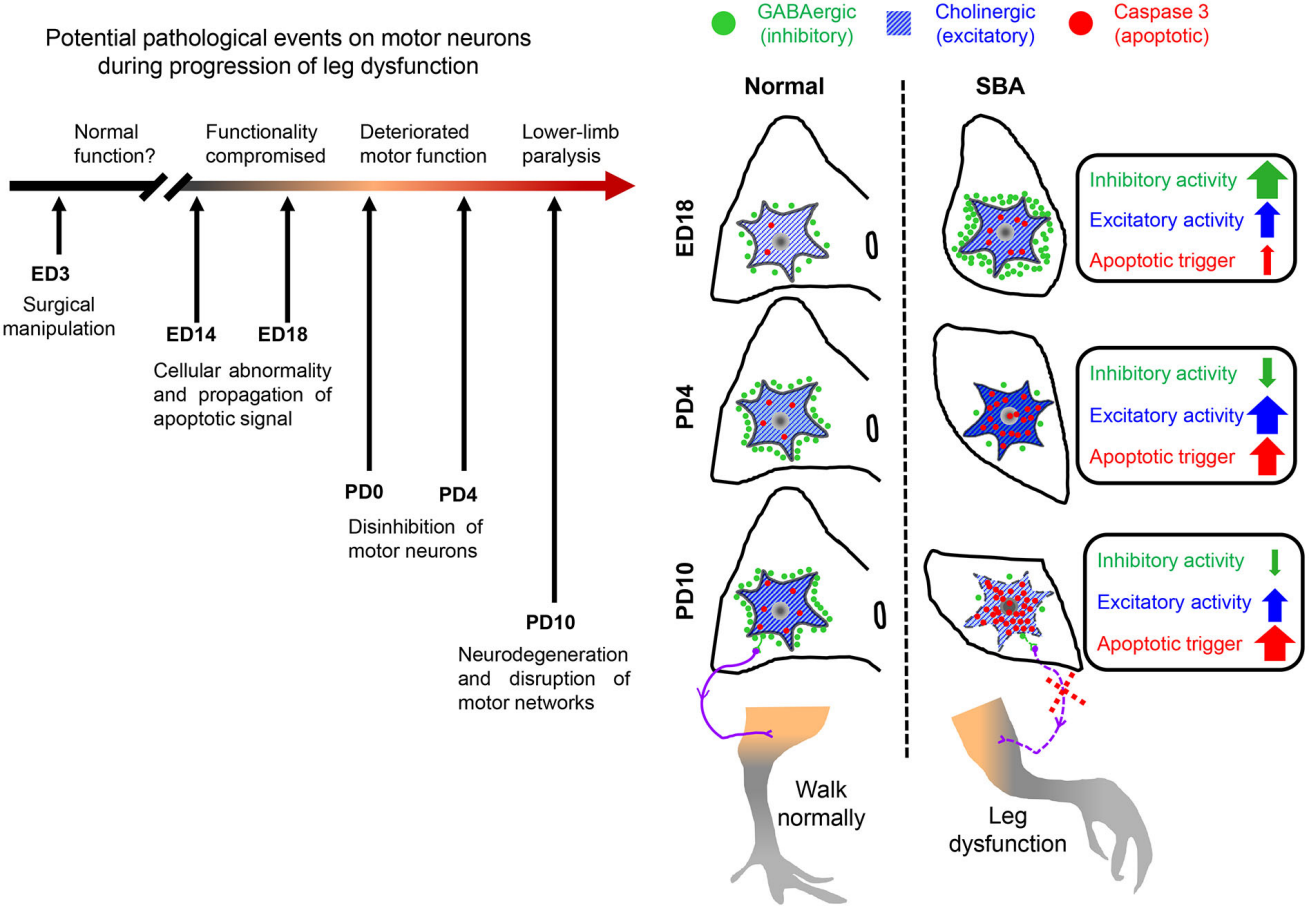
**Schematic of the pathological** **events in the spinal cord motor neurons during progression of SBA-like leg** **dysfunction.** The roof plate of the neural tube was incised on ED3 to expose the spinal cord to amniotic fluid. The trigger of cellular abnormalization (overexpression of inhibitory and excitatory transmission) and propagation of neurodegenerative signals (activation of caspase 3) were evident from mid- to advanced-gestational stage (ED14-ED18). At early neonatal life (PD0-PD4), the degree of SBA-like leg dysfunction correlated with the synaptic change-induced disinhibition of motor neurons in exposed lumbar cords, which were associated with a drastic loss of GABAergic transmission and the upregulation of cholinergic activities. At PD10, the SBA chicks not only exhibited drastic decreases in inhibitory tones on motor neurons but had also lost approximately half of their motor neurons via apoptosis, which is when the SBA chicks showed lower-limb paralysis.

Consistent with studies investigating human neonates with SBA, this study is the first to provide experimental evidence that the transiently present voluntary leg movements of SBA chicks disappear shortly after hatching. The degree of SBA-like motor dysfunction, particularly the loss of functionality in toe, ankle and knee joint movements, and the progression of lower-limb paralysis during early neonatal days (Fig. 1A, Table 1), were correlated with the synaptic change-induced disruption of motor networks in exposed lumbar cords (Figs 2 and 7). Furthermore, these changes were associated with drastic loss of GABAergic transmission and the upregulation of cholinergic activities (Figs 2 and 3; Fig. S2). Such loss of GABA might lead to increase excitatory drive to the motor neurons. The decrease in the number of inhibitory synaptic boutons that resulted in a lower ratio of inhibitory-to-excitatory inputs on the motor neurons (Fig. 2) provides further evidence of disinhibition of motor neurons, as do the deteriorated motor coordination (sit to walk), and rise of hyperreflexia and tremors function observed in SBA chicks during the early days of lower limb paralysis (Fig. 1A,B, Table 1). Therefore, it is possible that this type of disinhibition might have induced overexcitation of the motor neurons, which could have led to deteriorated motor function in SBA chicks in early neonatal life.

In addition, such disinhibition-induced overexcitation could have exacerbated existing excitotoxic events and accelerated the degeneration of synaptic boutons and motor neurons (Sunico et al., 2011; Ramírez-Jarquín et al., 2014) that lead to skeletal muscle weakness, disconnection of motor signals and paralysis of limbs (Schütz, 2005). The possibility of disconnected motor signals between spinal cords and leg muscles is also supported by the marked reduction in CB immunoreactivity in Renshaw cells (Fig. S3), which are the only GABAergic interneurons that receive afferents directly from motor neurons (Sanna et al., 1993; Ramírez-Jarquín et al., 2014). On PD10, SBA chicks not only exhibited drastic decreases in GABAergic transmission (Fig. 2C,viii,ix and Fig. 3,viii,xvi; Fig. S2,x,xx,xxx) and inhibitory synaptic boutons (Fig. 2B,i) on motor neurons, but had also lost approximately half of their motor neurons (Fig. 5B,vii), at which point the chicks completely lost their motor coordination and voluntary leg movements, and showed lower limb paralysis (Fig. 1A, Table 1). Taken together, these results indicate that the disinhibition-induced disruption of motor networks is a neuropathological hallmark contributing to enhanced SBA-like leg dysfunctions in the early neonatal days of SBA chicks. Our findings also strengthen the possibility of a correlation between the loss of GABAergic tones and motor dysfunction in SBA cases, because, as shown previously, intrathecal treatment with a GABA agonist relieves SBA-induced spasticity in humans (Bergenheim et al., 2003).

Qualitative deterioration of leg movements was evident from the first neonatal day in SBA chicks (Fig. 1A, Table 1), which has also been reported in human neonates with SBA (Sival et al., 2006), suggesting that the functional abnormalities in the spinal motor networks in these cases begin during the embryonic period. In our study, this was supported by the lower ratio of inhibitory-to-excitatory inputs to motor neurons in SBA chicks on PD0 (Fig. 2B,iii). Although the number of motor neurons seemed to be preserved in deformed spinal cords of SBA chicks during the gestational period (Fig. 5B), increases in GABAergic and cholinergic immunoreactivities were found in motor neurons at midgestation, and these activities were very strong at advanced stages (Fig. 2C and Fig. 3; Fig. S2). The widespread GABAergic immunoreactivity indicates that this neurotransmitter system might play a neuroprotective role against amniotic fluid-induced neurotoxicity and/or other neurotoxic insults including cholinergic-induced excitotoxicity, as has been observed in various nervous system tissues (Schütz, 2005; Nabeka et al., 2014). Alternatively, to maintain normal neurological functions during the gestational period, the increased levels of GABAergic transmissions might be necessary for sufficient inhibition of the increased cholinergic-induced excitation of motor neurons or vice versa (Ramírez-Jarquín et al., 2014). Moreover, the possibility that excess GABA might be a source of energy for abnormal neurons in exposed cords to promote proliferation and migration cannot be excluded (Neman et al., 2014; Van Swearingen et al., 2014). These possibilities are supported by our results, as SBA chicks had preserved interneurons (Fig. 4) and motor neurons (Fig. 5) with few apoptosis signals, along with enlarged areas of abnormal tissue (Fig. 1E) in advanced gestational stages. Although further studies are needed to clarify the actual roles that increased GABAergic and cholinergic activities play, our results indicate that the functionality of motor neurons in SBA chicks is compromised during the embryonic period, starting from at least midgestation.

The loss of neurons as a result of apoptosis is an important pathophysiological component of many neurological diseases (Tatton et al., 1997; Lawson and Lowrie, 1998; Keane et al., 2001; Scholz et al., 2005). The loss of spinal neurons at lesion sites might be associated with SBA-related neurological dysfunction (Adzick, 2012); however, little is known about the mechanism underlying neurodegeneration or the type of degenerated neurons that are associated with SBA-related motor dysfunction. In a previous study, we proposed that the loss of small neurons, which are most likely interneurons, in exposed spinal cords of SBA chicks at an early neonatal age is likely related to motor dysfunction (Mominoki et al., 2006). The present study provides evidence that apoptosis-induced degeneration of GABAergic interneurons and motor neurons in lumbar cords at lesion sites were associated with SBA-like motor dysfunctions (Figs 4 and 5). Kowitzke et al. (2016) also suggested that neurons in neonatal human myelomeningoceles cases could be degenerated via apoptosis, although Sival et al. (2008) was unable to detect apoptosis markers in motor neurons of autopsied spinal segments of human fetuses with SBA. This discrepancy might be caused by differences in the pathological stage of neurodegeneration because activation of caspase 3 is the final step required for the execution phase of apoptosis (Holtzman and Deshmukh, 1997). In this study, the immunoreactivity of caspase 3 in motor neurons of SBA chicks was evident in an advance gestational stage (ED18) but not at midgestation (ED14), which is when the cellular abnormalities were observed (Fig. S4), supporting the possibility of pathogenesis stage-dependent activation of caspase 3 in exposed spinal cords during embryonic development. The immunoreactivity of caspase 3 gradually increased with the progression of pathogenesis in early neonatal days and was very strong on PD10, which is when SBA chicks exhibited only a few damaged interneuron-like structures and approximately half the normal number of motor neurons (Figs 4 and 5).

Taken together, the present results indicate that apoptosis-induced degeneration of spinal neurons in lumbar cords are associated with SBA-like motor dysfunctions, and that these pathological changes occur within the first 2 weeks after hatching. However, the trigger of cellular abnormalization and propagation of neurodegenerative signals were evident from mid to advanced gestational stage, and those are likely a consequence of amniotic fluid-induced *in ovo* milieu, because mechanical and environmental insults in sham operated chicks had no, or very little, effect on neurodegeneration processes (Fig. 6; Figs S1 and S4). These results provide experimental evidence of the well-accepted ‘two-hit’ (Meuli et al., 1995a; Hutchins et al., 1996; Stiefel et al., 2007; Adzick, 2013), or recently proposed ‘three-hit’, hypothesis (Kowitzke et al., 2016) of neurodegeneration in SBA. Indeed, cellular abnormalization and changes in synaptic transmissions in motor neurons on ED14 (Fig. 3; Fig. S4) provide clues as to why the *in utero* closure of SBA lesions after midgestation cannot completely preserve motor function in humans with SBA (Tulipan et al., 1999; Sival et al., 2006). Collectively, the present findings demonstrate that the SBA-like leg dysfunction, reductions of inhibitory inputs to motor neurons, and increases in apoptotic activity have a causal relationship with the pathogenesis of motor disorders in SBA chicks (Fig. 7).

In conclusion, the present findings indicate that early postnatal loss of inhibitory transmission in the lumbar cord motor neurons might contribute to the pathophysiology of SBA-like leg dysfunction. Our results shed light on the mechanisms underlying the timing and causes of cellular abnormalization, disinhibition of motor neurons and neurodegeneration, thus opening up the possibility of new approaches to prevent neuronal function and degeneration in the progression of SBA-related motor complications. If our results are not unique to SBA chickens, thus also relevant in humans with SBA, our work should have a major impact on the development of novel therapeutic interventions for this congenital disease.

## MATERIALS AND METHODS

### Animals

The fertilized eggs of chickens (*Gallus gallus*; Mori Hatchery, Kagawa, Japan) were incubated in a commercial incubator (Showa Furanki, Saitama, Japan) at 37.8±0.2°C with 60% relative humidity to obtain embryos at developmental stages 17-21 inclusive on gestational day 2.5-3.5. The developmental stage of each embryo was determined using the developmental table of Hamburger and Hamilton (1951). The embryos were then divided into two groups: the SBA group, in which the roof plate of the neural tube was incised, and the normal control group, in which the neural tube was left intact. The hatched chicks were raised in a room kept at 30°C with continuous lighting and fed a commercial diet (crude protein, 24%; metabolizable energy, 3050 kcal/kg; Toyohashi Feed Mills, Aichi, Japan) with water available *ad libitum*. Because the SBA chicks were unable to eat food by themselves during early neonatal days, they were gavaged a feed slurry (tube feeding) at a mass of 4.0% body weight into the crop (four times in a day). The feed slurry was made by mixing 40% powdered diet with 60% distilled water on a weight basis. The animal experimental protocols were approved by the Committee on the Ethics of Animal Experiments of the Ehime University Graduate School of Medicine, Japan (05A27-10).

### Surgical manipulation for generating SBA chicks

To produce SBA chicks, surgical manipulation of the neural plate was carried out according to a previously described procedure (Mominoki et al., 2006). Briefly, after removal of ∼1 ml of albumin, the egg shell and amnion were opened and placed under a stereomicroscope to determine the developmental stage of the embryo. Then, the roof plate of the neural tube was incised longitudinally, starting at the level of the cranial margin of the 26th somite, which forms the sixth and seventh thoracic segments, using a custom-made microknife (Kinutani and Le Douarin, 1985; Kinutani et al., 1986). The incision extended caudally for a distance equivalent to the length of seven somites, and was made by inserting the microknife into the neural tube to approximately half the depth of the tube. The roof plate was incised with care taken not to damage other parts of the neural tube. After the incision was made and the surgical manipulation was complete, the shell window was closed using transparent adhesive tape and the eggs were reincubated at 37.8±0.2°C with 60% relative humidity.

### Motor behavior and neurological assessments

The induction of SBA in the chicks was confirmed by observing open defects on the backs of the chicks with leg dysfunction (Fig. S1D). For motor behavior and neurological assessments, the chicks (SBA and control) used at P0 were also used at P2, P4 and P10. To characterize the leg movements of the chicks, the spontaneous locomotor activities of the normal and SBA chicks were videotaped at 08:00 and 20:00 for 10 min at each evaluation age (PD0, PD2, PD4 and PD10) and scored offline by two independent observers. The bilateral leg movements were categorized following assessments of movement at each joint (hip, knee, ankle and toes) for each leg as follows: no movement (0 points), slight movement (1-2 points; defined as movement of the joint that was less than half of full range), reduced movement (3-4 points, defined as movement of the joint that was greater than or equal to half of full range) and normal movement (5 points). Motor dysfunction was evaluated by assessing the ability to sit-stand-walk as follows: unable (0 points), minimal (1-2 points), reduced (3-4 points), and normal (5 points). Additionally, the total number of leg movements (stepping and/or leg flapping) per 10 min were assessed in the SBA and normal chicks. There were six chicks in each group at each age point for behavioral analyses.

### Sample collection and tissue preparation

Spinal cord sections from the lumbosacral region were collected from the normal and SBA chicks on ED14, ED18, PD2, PD4 and PD10. The incisions of the roof plate of the neural tube in the SBA embryos were confirmed by observing open defects on the backs of the chicks (data not shown). Six chicks from each group at each age point were transcardially perfused with a fixative solution containing 4% paraformaldehyde with 0.5% glutaraldehyde in 0.1 M phosphate-buffered saline (PBS). After the perfusion fixation, the spinal cord was removed from the location of open defect (exposed area, lesion location) (Fig. 1C,ii). Spinal tissue samples from the normal control chicks were collected from location that was similar to those of the SBA chicks. Following the sample collection, the tissues were immersed in 4% paraformaldehyde overnight at 4°C. Next, the samples were dehydrated and embedded in paraffin.

For the electron microscopy analysis, three chicks from each group on PD0, PD2, PD4 and PD10 were transcardially perfused using the procedures described above, except that 3% glutaraldehyde was used instead of 0.5% glutaraldehyde. Following the perfusion, spinal cord samples were collected from the location of the open defect in the SBA chicks and from a similar location in the normal control chicks. After a rinse in 0.2 M phosphate buffer and postosmification, the samples were stained en bloc with a saturated aqueous uranyl acetate solution for 2 h, dehydrated in a graded ethanol series, and then embedded in Epon epoxy resin (Hexion, Columbus, OH, USA).

### Surgical manipulation for generating sham control chicks

To clarify whether the mechanical and environmental insults altered the spinal motor networks, two types of sham injury control groups were created. In the first group, the embryos were treated in the same manner as the SBA group, except that the length of incision in the roof plate was less than three somites (ES-sham; Fig. S1C). The incidence of open neural tube defect and leg dysfunctions in survivors of ES-sham group was 0% in this study (Fig. S1C) and a previous study (Mominoki et al., 2006). In the second group, a laminectomy was performed at three vertebral segments, L2-L4, to make an SBA-like open defect (∼1 cm) in the backs of the chicks (PS-sham; Fig. S1B). Briefly, the 1-day-old (6-10 h after hatching) normal chicks were deeply anesthetized with an intraperitoneal injection of sodium pentobarbital (0.5 mg/kg), and anesthesia was verified by the absence of both the leg withdrawal reflex to a toe pinch and the corneal blink reflex. After shaving and cleansing the surgical field, the skin of the back was incised, the underlying muscles were retracted to expose the lumbar vertebrae, and a total laminectomy was performed under a microscope; special care was taken not to injure the spinal cord and to keep the dura intact. Following the surgical procedure, the chicks were placed in a heating chamber and their body temperatures were maintained at ∼37°C until fully awake, when they were returned to their cages. Using the methods described earlier, spinal cord samples were collected from a similar location in the lumbar cord for both sham groups on ED14, ED18, PD4 and PD10; there were four chicks in each group at each age point.

### Histopathological analysis

Serial coronal sections (7 µm) from the location of the open defect (exposed area) of the lumbar cord in the SBA chicks, and from a similar location in the normal chicks (Fig. 1C), were stained with Hematoxylin-Eosin. The tissues were observed under a Nikon Eclipse E800 light microscope (Nikon, Tokyo, Japan), images were acquired using a digital camera attached to the microscope (Nikon Digital Sight DS-L2), and the total cross-sectional and gray matter areas were determined using ImageJ software (https://imagej.nih.gov/ij/). The tissue sections were treated with a Nissl stain (Cresyl Violet) and the total number of motor neurons was quantified by a blind rater. The ventral horn was defined as gray matter ventral to the central canal and only motor neurons with a clearly identifiable nucleus were counted. The total tissue area, gray matter surface area and number of motor neurons were calculated and averaged across six cross sections per chick at each age point; there were six chicks in each group at each age point.

To assess the presence of neuropathological alterations in motor neuron area, the tissue embedded in resin for the electron microscopy analyses was used for light microscopy analyses. Semi-thin sections (0.5 µm) were cut using an ultramicrotome (Leica VIM-535; Reichert-Nissei Ultracuts, Vienna, Austria), collected on a slide, and then lightly stained with a solution containing 1% Toluidine Blue and 1% borax in distilled water. The tissue was covered with a Toluidine Blue solution, incubated for 2-5 min on a heater at 60°C, rinsed with distilled water, and then air-dried. Finally, it was observed under a light microscope (Nikon).

### Electron microscopy

For electron microscopy, a rectangular area of the lateral ventral horn was removed; ultrathin sections (80 nm) were made with a diamond knife and mounted on single-slot grids coated with Formvar film. The double-stained specimens stained with uranyl acetate and lead citrate were examined with an electron microscope (JEM-1230, 80kV; JEOL USA, Peabody, MA, USA). Electron microscopic profiles were identified as synaptic boutons only if the profile had all of the characteristics of synaptic structures with respect to synaptic vesicles, synaptic density and typical membrane apposition. Classification and quantification of the boutons were conducted according to the type of synaptic vesicle, as previously described: synaptic boutons were classified as S-boutons if >80% of the vesicles were spherical, while those that contained >20% flat vesicles were classified as F-boutons, because the S-type (Fig. 2A, asterisks) or F-type (Fig. 2A, hash signs) vesicles are presumably excitatory and inhibitory synapses, respectively (Uchizono, 1965; Matsuda et al., 2004; Sunico et al., 2011). Approximately 2% of the boutons were omitted from further morphometric or statistical analyses because they contained only a few vesicles or were irregularly shaped. To be considered for the analysis of synaptic boutons, each motor neuron was required to have 18-22 electron micrographs, obtained at 20,000× magnification. The numbers of each type of synaptic boutons on the motor neurons were calculated and averaged across 10 motor neurons in each chicks at each age point. Furthermore, the total number of synaptic boutons (both inhibitory and excitatory) and the ratio of inhibitory-to-excitatory boutons per motor neuron was calculated and averaged in each chick at each age point. There were three chicks in each group at each age point.

### Immunofluorescence staining

Immunofluorescence staining was performed as described previously (Nabeka et al., 2014). The sections were incubated with one of the following primary antibodies for 60 h at 4°C: rabbit polyclonal anti-GABA (1:3000; Sigma, St. Louis, MO, USA), mouse monoclonal anti-GAD67 (1:500; Millipore, Temecula, CA, USA), mouse monoclonal anti-CB (1:500; SWANT, Bellinzona, Switzerland), mouse monoclonal anti-CR (1:500; SWANT, Bellinzona, Switzerland), rabbit polyclonal anti-ChAT (1:500; Abbiotec, San Diego, CA, USA) or rabbit polyclonal anti-caspase 3 (1:500; Bioss, Woburn, MA, USA). After a wash in PBS, the sections were treated for 2 h at room temperature with either Alexa Fluor 546 goat anti-rabbit IgG (H+L) (1:1000; Invitrogen, Carlsbad, CA, USA) or Alexa Fluor 488 goat anti-mouse IgG (H+L) (1:1000; Invitrogen) and DAPI. The sections were then washed with PBS, mounted in Vectashield (Vector Laboratories, Burlingame, CA, USA), and examined in high-resolution confocal images obtained using a Nikon A1 confocal microscope equipped with a 100× objective lens (Nikon).

For double immunofluorescence staining, the sections were incubated for 60 h in a solution containing rabbit polyclonal anti-GABA (1:3000; Sigma-Aldrich) plus mouse monoclonal anti-CB or mouse monoclonal anti-CR (1:500; SWANT); and rabbit polyclonal anti-caspase 3 (1:500; Bioss) plus mouse monoclonal anti-GAD 67 (1:500, Millipore) or mouse monoclonal anti-CB (1:500; SWANT). After washing in PBS, the sections were treated for 2 h at room temperature with either Alexa Fluor 546 goat anti-rabbit IgG (H+L) (1:1000; Invitrogen) or Alexa Fluor 488 goat anti-mouse IgG (H+L) (1:1000; Invitrogen) and DAPI, then washed with PBS, mounted in Vectashield (Vector Laboratories), and examined in high-resolution confocal images obtained using a Nikon A1 confocal microscope equipped with a 100× objective lens (Nikon).

The specificities of the antibody staining were tested using a negative staining procedure with normal rabbit IgG instead of the primary antibodies; the samples were processed as described above. Nonspecific staining was not observed (data not shown).

### Staining intensity measurement

The staining intensities of GABA, GAD67 and ChAT in the ventral horn (motor neuron area) of the open defect lumbar cord in SBA chicks, and in a similar location in normal chicks, at different age points were measured using ImageJ software. To correct for background in images, three background intensity readings were taken per image. These readings were subsequently averaged and subtracted from the signal intensity around motor neurons (for GABA and GAD67) or within motor neurons (for ChAT) to give an accurate reading of the candidate protein staining intensity. The intensity data were presented as mean±s.e.m. Six random sections per chick at each age point in each group were analyzed for quantification; there were six chicks in each group at each age point.

### Statistical analysis

All quantitative parameters (stepping/leg flapping, tissue area, number of synaptic boutons, immunoreactive intensities and number of motor neurons) were quantified and compared between the normal control and SBA groups, except if mentioned otherwise. Statistics were performed using the average values and all data are reported as mean±s.e.m. The data were analyzed with two-way analyses of variance (ANOVA) and Tukey–Kramer post hoc comparisons, and *P*<0.05 was considered to indicate statistical significance.

## Supplementary Material

Supplementary information

## References

[DMM031054C1] Adzick N. S. (2012). Fetal surgery for myelomeningocele: trials and tribulations. Isabella Forshall Lecture. *J. Pediatr. Surg.* 47, 273-281. 10.1016/j.jpedsurg.2011.11.021 22325376 PMC3278714

[DMM031054C2] Adzick N. S. (2013). Fetal surgery for spina bifida: past, present, future. *Semin. Pediatr. Surg.* 22, 10-17. 10.1053/j.sempedsurg.2012.10.003 23395140 PMC6225063

[DMM031054C3] Adzick N. S., Sutton L. N., Crombleholme T. M. and Flake A. W. (1998). Successful fetal surgery for spina bifida. *Lancet* 352, 1675-1676. 10.1016/S0140-6736(98)00070-1 9853442

[DMM031054C4] Alles A. J. and Sulik K. K. (1990). Retinoic acid-induced spina bifida: evidence for a pathogenetic mechanism. *Development* 108, 73-81. 2190788 10.1242/dev.108.Supplement.73

[DMM031054C5] Bergenheim A. T., Wendelius M., Shahidi S. and Larsson E. (2003). Spasticity in a child with myelomeningocele treated with continuous intrathecal baclofen. *Pediatr. Neurosurg.* 39, 218-221. 10.1159/000072476 12944705

[DMM031054C6] Copp A. J., Seller M. J. and Polani P. E. (1982). Neural tube development in mutant (curly tail) and normal mouse embryos: the timing of posterior neuropore closure in vivo and in vitro. *J. Embryol. Exp. Morphol.* 69, 151-167. 7119666

[DMM031054C7] Copp A. J., Brook F. A., Estibeiro J. P., Shum A. S. W. and Cockroft D. L. (1990). The embryonic development of mammalian neural tube defects. *Prog. Neurobiol.* 35, 363-403. 10.1016/0301-0082(90)90037-H 2263736

[DMM031054C8] Copp A. J., Adzick N. S., Chitty L. S., Fletcher J. M., Holmbeck G. N. and Shaw G. M. (2015). Spina bifida. *Nat. Rev. Dis. Primers* 1, 15007 10.1038/nrdp.2015.7 27189655 PMC4898641

[DMM031054C9] Danzer E., Schwarz U., Wehrli S., Radu A., Adzick N. S. and Flake A. W. (2005). Retinoic acid induced myelomeningocele in fetal rats: characterization by histopathological analysis and magnetic resonance imaging. *Exp. Neurol.* 194, 467-475. 10.1016/j.expneurol.2005.03.011 15893307

[DMM031054C10] Fichter M. A., Dornseifer U., Henke J., Schneider K. T. M., Kovacs L., Biemer E., Bruner J., Adzick N. S., Harrison M. R. and Papadopulos N. A. (2008). Fetal spina bifida repair--current trends and prospects of intrauterine neurosurgery. *Fetal Diagn. Ther.* 23, 271-286. 10.1159/000123614 18417993

[DMM031054C11] Gottesfeld Z., Teitelbaum D., Webb C. and Arnon R. (1976). Changes in the GABA system in experimental allergic encephalomyelitis-induced paralysis. *J. Neurochem.* 27, 695-699. 10.1111/j.1471-4159.1976.tb10396.x 966011

[DMM031054C12] Hamburger V. and Hamilton H. L. (1951). A series of normal stages in the development of the chick embryo. *J. Morphol.* 88, 49-92. 10.1002/jmor.1050880104 24539719

[DMM031054C13] Heffez D. S., Aryanpur J., Hutchins G. M. and Freeman J. M. (1990). The paralysis associated with myelomeningocele: clinical and experimental data implicating a preventable spinal cord injury. *Neurosurgery* 26, 987-992. 10.1227/00006123-199006000-00011 2362676

[DMM031054C14] Heffez D. S., Aryanpur J., Rotellini N. A. C., Hutchins G. M. and Freeman J. M. (1993). Intrauterine repair of experimental surgically created dysraphism. *Neurosurgery* 32, 1005-1010. 10.1227/00006123-199306000-00021 8327074

[DMM031054C15] Holtzman D. M. and Deshmukh M. (1997). Caspases: a treatment target for neurodegenerative disease? *Nat. Med.* 3, 954-955. 10.1038/nm0997-954 9288715

[DMM031054C16] Hutchins G. M., Meuli M., Meuli-Simmen C., Jordan M. A., Heffez D. S. and Blakemore K. J. (1996). Acquired spinal cord injury in human fetuses with myelomeningocele. *Pediatr. Pathol. Lab. Med.* 16, 701-712. 10.1080/15513819609169297 9025869

[DMM031054C17] Keane R. W., Kraydieh S., Lotocki G., Alonso O. F., Aldana P. and Dietrich W. D. (2001). Apoptotic and antiapoptotic mechanisms after traumatic brain injury. *J. Cereb. Blood Flow Metab.* 21, 1189-1198. 10.1097/00004647-200110000-00007 11598496

[DMM031054C18] Kinutani M. and Le Douarin N. M. (1985). Avian spinal cord chimeras: I. Hatching ability and posthatching survival in homo- and heterospecific chimeras. *Dev. Biol.* 111, 243-255. 10.1016/0012-1606(85)90449-X 4029508

[DMM031054C19] Kinutani M., Coltey M. and Le Douarin N. M. (1986). Postnatal development of a demyelinating disease in avian spinal cord chimeras. *Cell* 45, 307-314. 10.1016/0092-8674(86)90395-8 3698100

[DMM031054C20] Korenromp M. J., van Gool J. D., Bruinese H. W. and Kriek R. (1986). Early fetal leg movements in myelomeningocele. *Lancet* 1, 917-918. 10.1016/S0140-6736(86)91022-6 2870386

[DMM031054C21] Kowitzke B., Cohrs G., Leuschner I., Koch A., Synowitz M., Mehdorn H. M., Held-Feindt J. and Knerlich-Lukoschus F. (2016). Cellular profiles and molecular mediators of lesion cascades in the placode in human open spinal neural tube defects. *J. Neuropathol. Exp. Neurol.* 75, 827-842. 10.1093/jnen/nlw057 27354486

[DMM031054C22] Lawson S. J. and Lowrie M. B. (1998). The role of apoptosis and excitotoxicity in the death of spinal motoneurons and interneurons after neonatal nerve injury. *Neuroscience* 87, 337-348. 10.1016/S0306-4522(98)00120-1 9740396

[DMM031054C23] Matsuda S., Kobayashi Y. and Ishizuka N. (2004). A quantitative analysis of the laminar distribution of synaptic boutons in field CA3 of the rat hippocampus. *Neurosci. Res.* 49, 241-252. 10.1016/j.neures.2004.03.002 15140566

[DMM031054C24] Meuli M., Meuli-Simmen C., Hutchins G. M., Yingling C. D., Hoffman K. M. B., Harrison M. R. and Adzick N. S. (1995a). In utero surgery rescues neurological function at birth in sheep with spina bifida. *Nat. Med.* 1, 342-347. 10.1038/nm0495-342 7585064

[DMM031054C25] Meuli M., Meuli-Simmen C., Yingling C. D., Hutchins G. M., Hoffman K. M. B., Harrison M. R. and Adzick N. S. (1995b). Creation of myelomeningocele in utero: a model of functional damage from spinal cord exposure in fetal sheep. *J. Pediatr. Surg.* 30, 1028-1033. 10.1016/0022-3468(95)90335-6 7472926

[DMM031054C26] Meuli M., Meuli-Simmen C., Yingling C. D., Hutchins G. M., Timmel G. B., Harrison M. R. and Adzick N. S. (1996). In utero repair of experimental myelomeningocele saves neurological function at birth. *J. Pediatr. Surg.* 31, 397-402. 10.1016/S0022-3468(96)90746-0 8708911

[DMM031054C27] Meuli M. and Moehrlen U. (2014). Fetal surgery for myelomeningocele is effective: a critical look at the whys. *Ped. Surg. Int.* 30, 689-697. 10.1007/s00383-014-3524-8 24908159

[DMM031054C28] Millicovsky G. and Lazar M. L. (1995). Spina bifida: role of neural tissue damage during pregnancy in producing spinal paralysis. *Obstet. Gynecol.* 86, 300-301. 10.1016/0029-7844(95)00141-D 7617367

[DMM031054C29] Mominoki K., Kinutani M., Wakisaka H., Saito S., Kobayashi N., Fujiwara T. and Matsuda, S. (2006). Leg dysfunctions in a hatched chick model of spina bifida aperta. *Exp. Neurol.* 197, 133-142. 10.1016/j.expneurol.2005.09.001 16203002

[DMM031054C30] Nabeka H., Uematsu K., Takechi H., Shimokawa T., Yamamiya K., Li C., Doihara T., Saito S., Kobayashi N. and Matsuda S. (2014). Prosaposin overexpression following kainic acid-induced neurotoxicity. *PLoS ONE* 9, e110534 10.1371/journal.pone.0110534 25461957 PMC4251898

[DMM031054C31] Neman J., Termini J., Wilczynski S., Vaidehi N., Choy C., Kowolik C. M., Li H., Hambrecht A. C., Roberts E. and Jandial R. (2014). Human breast cancer metastases to the brain display GABAergic properties in the neural niche. *Proc. Natl. Acad. Sci. USA* 111, 984-989. 10.1073/pnas.1322098111 24395782 PMC3903266

[DMM031054C32] Nikolopoulou E., Galea G. L., Rolo A., Greene N. D. E. and Copp A. J. (2017). Neural tube closure: cellular, molecular and biomechanical mechanisms. *Development* 144, 552-566. 10.1242/dev.145904 28196803 PMC5325323

[DMM031054C33] Osaka K., Tanimura T., Hirayama A. and Matsumoto S. (1978). Myelomeningocele before birth. *J. Neurosurg.* 49, 711-724. 10.3171/jns.1978.49.5.0711 712393

[DMM031054C34] Papanna R., Mann L. K., Snowise S., Morales Y., Prabhu S. P., Tseng S. C., Grill R., Fletcher S. and Moise K. J. Jr (2016). Neurological outcomes after human umbilical cord patch for in utero spina bifida repair in a sheep model. *AJP Rep.* 6, e309-e317. 10.1055/s-0036-1592316 27621952 PMC5017885

[DMM031054C35] Ramírez-Jarquín U. N., Lazo-Gómez R., Tovar-Y-Romo L. B. and Tapia R. (2014). Spinal inhibitory circuits and their role in motor neuron degeneration. *Neuropharmacology* 82, 101-107. 10.1016/j.neuropharm.2013.10.003 24157492

[DMM031054C36] Sanna P. P., Celio M. R., Bloom F. E. and Rende M. (1993). Presumptive Renshaw cells contain decreased calbindin during recovery from sciatic nerve lesions. *Proc. Natl. Acad. Sci. USA* 90, 3048-3052. 10.1073/pnas.90.7.3048 8464922 PMC46234

[DMM031054C37] Scholz J., Broom D. C., Youn D. H., Mills C. D., Kohno T. and Suter M. R., Moore K. A., Decosterd I., Coggeshall R. E. and Woolf C. J. (2005). Blocking caspase activity prevents transsynaptic neuronal apoptosis and the loss of inhibition in lamina II of the dorsal horn after peripheral nerve injury. *J. Neurosci.* 25, 7317-7323. 10.1523/JNEUROSCI.1526-05.2005 16093381 PMC6725303

[DMM031054C38] Schütz B. (2005). Imbalanced excitatory to inhibitory synaptic input precedes motor neuron degeneration in an animal model of amyotrophic lateral sclerosis. *Neurobiol. Dis.* 20, 131-140. 10.1016/j.nbd.2005.02.006 16137574

[DMM031054C39] Selçuki M., Manning S. and Bernfield M. (2001). The curly tail mouse model of human neural tube defects demonstrates normal spinal cord differentiation at the level of the meningomyelocele: implications for fetal surgery. *Child's Nerv. Syst.* 17, 19-23. 10.1007/s003810000401 11219618

[DMM031054C40] Sim K.-B., Lee J. Y., Phi J. H., Kim S.-K. and Wang K.-C. (2013). Experimental models of spinal open neural tube defect and Chiari type II malformation. *Childs Nerv. Syst.* 29, 1435-1449. 10.1007/s00381-013-2148-y 24013317

[DMM031054C41] Sinnamon H. M. and Benaur M. (1997). GABA injected into the anterior dorsal tegmentum (ADT) of the midbrain blocks stepping initiated by stimulation of the hypothalamus. *Brain Res.* 766, 271-275. 10.1016/S0006-8993(97)00734-8 9359615

[DMM031054C42] Sival D. A., Begeer J. H., Staal-Schreinemachers A. L., Vos-Niël J. M., Beekhuis J. R. and Prechtl H. F. R. (1997). Perinatal motor behaviour and neurological outcome in spina bifida aperta. *Early Hum. Dev.* 50, 27-37. 10.1016/S0378-3782(97)00090-X 9467691

[DMM031054C43] Sival D. A., van Weerden T. W., Vles J. S. H., Timmer A., den Dunnen W. F. A., Staal-Schreinemachers A. L., Hoving E. W., Sollie K. M., Kranen-Mastenbroek V. J. M., Sauer P. J. J. et al. (2004). Neonatal loss of motor function in human spina bifida aperta. *Pediatrics* 114, 427-434. 10.1542/peds.114.2.427 15286226

[DMM031054C44] Sival D. A., Brouwer O. F., Bruggink J. L. M., Vles J. S. H., Staal-Schreinemachers A. L., Sollie K. M., Sauer P. J. J. and Bos A. F. (2006). Movement analysis in neonates with spina bifida aperta. *Early Hum. Dev.* 82, 227-234. 10.1016/j.earlhumdev.2005.09.002 16256280

[DMM031054C45] Sival D. A., Verbeek R. J., Brouwer O. F., Sollie K. M., Bos A. F. and den Dunnen W. F. A. (2008). Spinal hemorrhages are associated with early neonatal motor function loss in human spina bifida aperta. *Early Hum. Dev.* 84, 423-431. 10.1016/j.earlhumdev.2007.11.003 18180116

[DMM031054C46] Stiefel D., Copp A. J. and Meuli M. (2007). Fetal spina bifida in a mouse model: loss of neural function in utero. *J. Neurosurg.* 106, 213-221. 10.3171/ped.2007.106.3.213 17465388 PMC3651953

[DMM031054C47] Sunico C. R., Domínguez G., García-Verdugo J. M., Osta R., Montero F. and Moreno-López B. (2011). Reduction in the motoneuron inhibitory/excitatory synaptic ratio in an early-symptomatic mouse model of amyotrophic lateral sclerosis. *Brain Pathol.* 21, 1-15. 10.1111/j.1750-3639.2010.00417.x 20653686 PMC8094302

[DMM031054C48] Tatton W. G., Chalmers-Redman R. M. E., Ju W. Y. H., Wadia J. and Tatton N. A. (1997). Apoptosis in neurodegenerative disorders: potential for therapy by modifying gene transcription. *J. Neural. Transm. Suppl.* 49, 245-268. 10.1007/978-3-7091-6844-8_25 9266433

[DMM031054C49] Tsujimura R., Mominoki K., Kinutani M., Shimokawa T., Doihara T., Nabeka H., Wakisaka H., Kobayashi N. and Matsuda S. (2011). Sensory tract abnormality in the chick model of spina bifida. *Neurosci. Res.* 71, 85-91. 10.1016/j.neures.2011.05.017 21658418

[DMM031054C50] Tulipan N., Bruner J. P., Hernanz-Schulman M., Lowe L. H., Walsh W. F., Nickolaus D. and Oakes W. J. (1999). Effect of intrauterine myelomeningocele repair on central nervous system structure and function. *Pediatr. Neurosurg.* 31, 183-188. 10.1159/000028859 10705927

[DMM031054C51] Uchizono K. (1965). Characteristics of excitatory and inhibitory synapses in the central nervous system of the cat. *Nature* 207, 642-643. 10.1038/207642a0 5883646

[DMM031054C52] Van Swearingen A. E., Siegel M. B. and Anders C. K. (2014). Breast cancer brain metastases: evidence for neuronal-like adaptation in a ‘breast-to-brain’ transition? *Breast Cancer Res.* 16, 304 10.1186/bcr3651 25679873 PMC4052941

[DMM031054C53] Watanabe M., Li H., Kim A. G., Weilerstein A., Radu A., Davey M., Loukogeorgakis S., Sánchez M. D., Sumita K., Morimoto N. et al. (2016). Complete tissue coverage achieved by scaffold-based tissue engineering in the fetal sheep model of Myelomeningocele. *Biomaterials* 76, 133-143. 10.1016/j.biomaterials.2015.10.051 26520044

