# Supplementary material for: Early neonatal loss of inhibitory synaptic input to the spinal motor neurons confers spina bifida-like leg dysfunction in a chicken model
Source: Dis Model Mech. 2017 Dec 1;10(12):1421–32. doi: 10.1242/dmm.031054 (PMC5769610; doi:10.1242/dmm.031054)
Supplement: Supplementary information [file dmm-10-031054-s1.pdf]

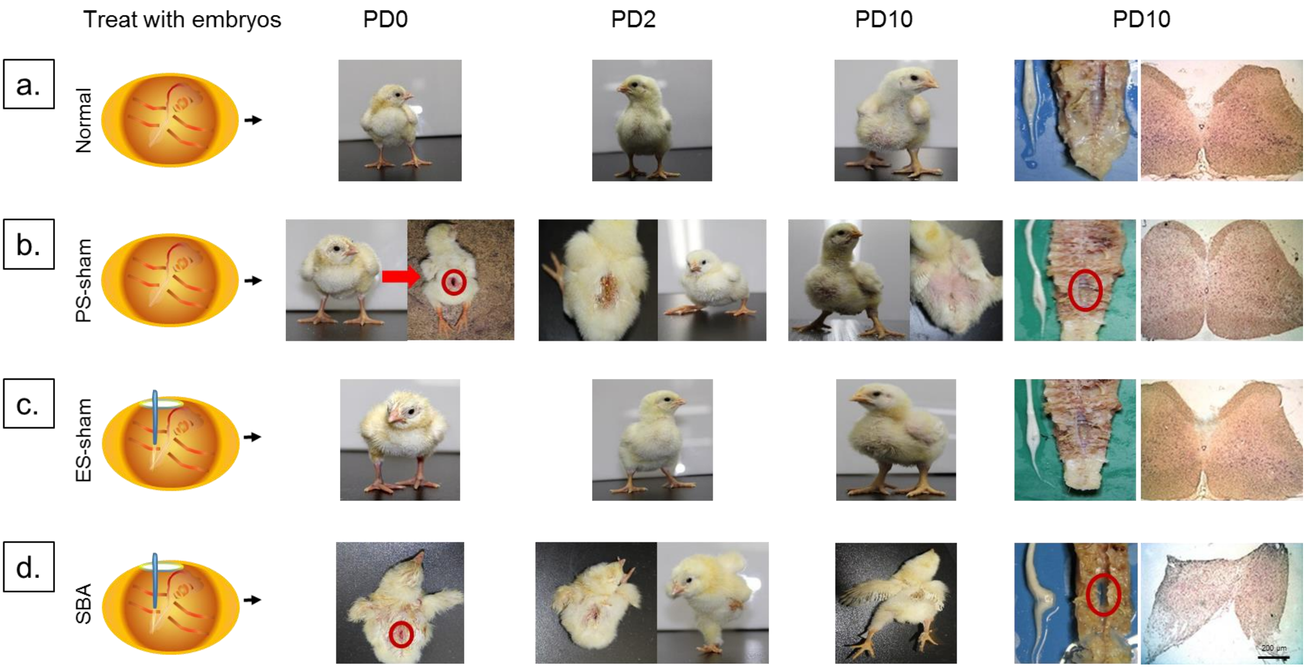

Supplementary Figure S1. Schematic depicting the process used to generate SBA and sham-control chicks. (a) Normal control group: chicks from intact eggs were used as the normal controls. (b) PS-sham group: an SBA-like open defect was made in the lumbosacral area via a laminectomy at three vertebral segments (L2–L4) in normal chicks at P-0. (c) ES-sham group: embryos underwent the same surgical manipulation as the SBA chicks, except that the length of incision in the roof plate was shorter than three somites. (d) SBA group: the length of incision in the roof plate was longer than five somites. Although PS-sham chicks showed reduced ability to walk during the first 2 days after laminectomy, their voluntary leg movements on PD10 remained functional. Neither sham group showed spinal cord deformation or changes in tissue area at the lesion sites.

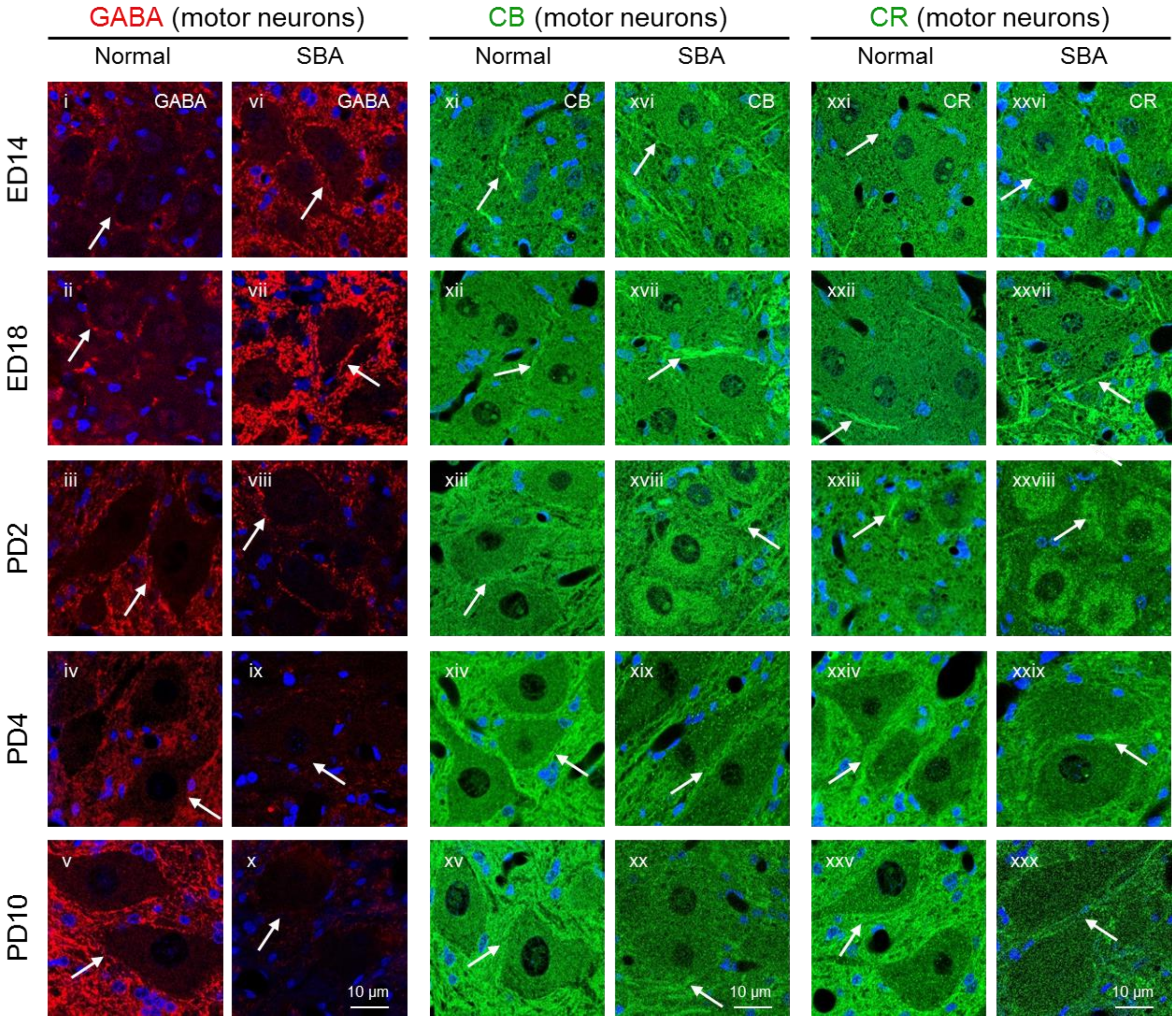

Supplementary Figure S2. Analysis of GABAergic immunoreactivities on motor neurons in spinal cord of normal and SBA chicks. Immunofluorescence staining was performed with antibodies directed against GABA (i–x) or calbindin-D-28K (CB; xi–xx) or calretinin (CR; xxi–xxx). Representative confocal images from the ventral horn of the open defect in the lumbar cord of SBA chicks and in a similar location in normal controls at ED14, ED18, PD2, PD4, and PD10. GABA (i–v; arrows), CB (xi–xv; arrows), or CR (xxi–xxv; arrows) immunoreactivity around the lumbar cord motor neurons increased in normal chicks as the developmental stage increased.

Comparing with control chicks, very strong GABA (vi and vii; arrows), CB (xvi and xvii; arrows), or CR (xxvi and xxvii; arrows) labeling was observed around the motor neurons of the SBA chicks on ED14 and ED18 but decreased by the post-hatch ages; in particular, there was a drastic decrease in, or almost complete absence of, GABA (x; arrows), CB (xx; arrows), or CR (xxx; arrows) labeling around the motor neurons in the exposed cord area in SBA chicks on PD10.

Disease Models & Mechanisms • Supplementary information

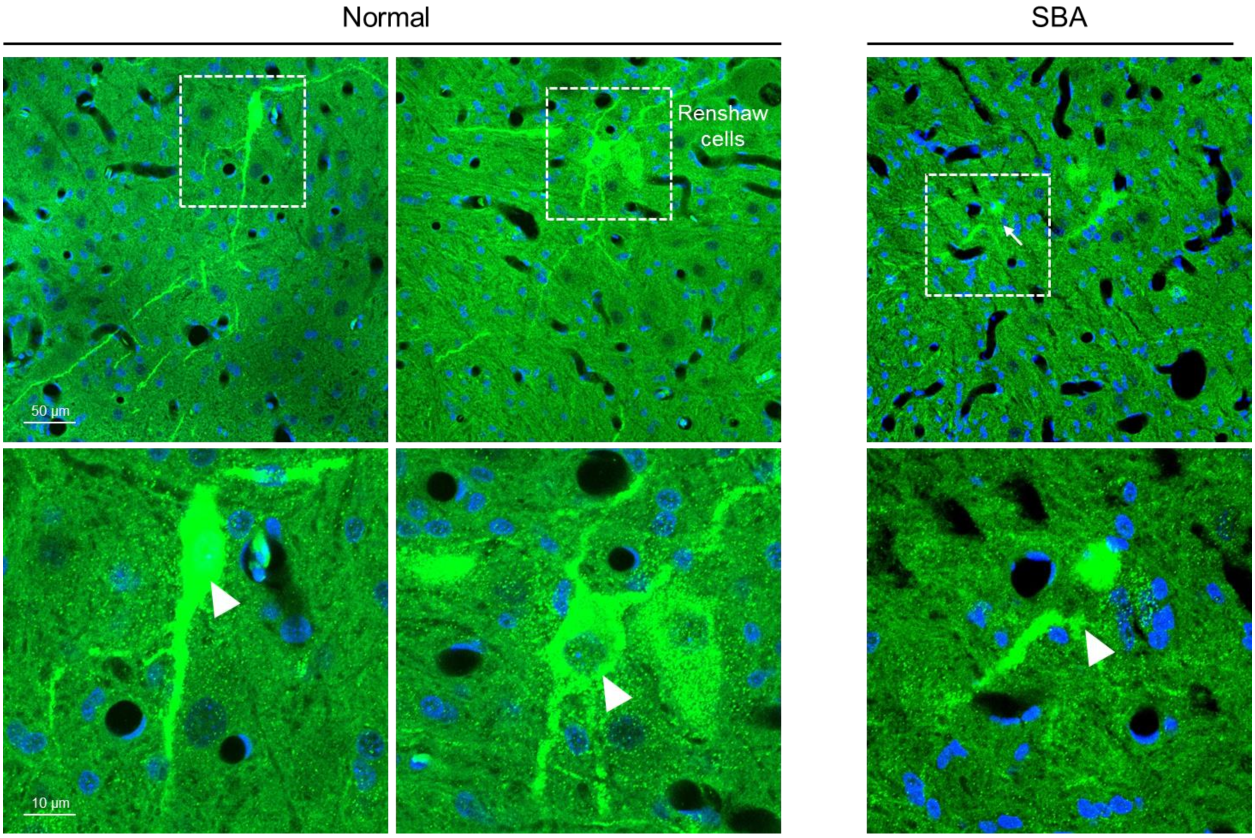

Supplementary Figure S3. Analysis of CB immunoreactivity in Renshaw cells in spinal cord of normal and SBA chicks. Representative confocal images showing calbindin-D-28K (CB) immunoreactivity in the Renshaw cells of normal (a–d) and SBA (e and f) chicks at PD4. The immunoreactivities for CB and DAPI are shown in green and blue, respectively. The intensity of CB immunoreactivity in Renshaw cells (arrowheads) was weaker in SBA chicks relative to normal chicks.

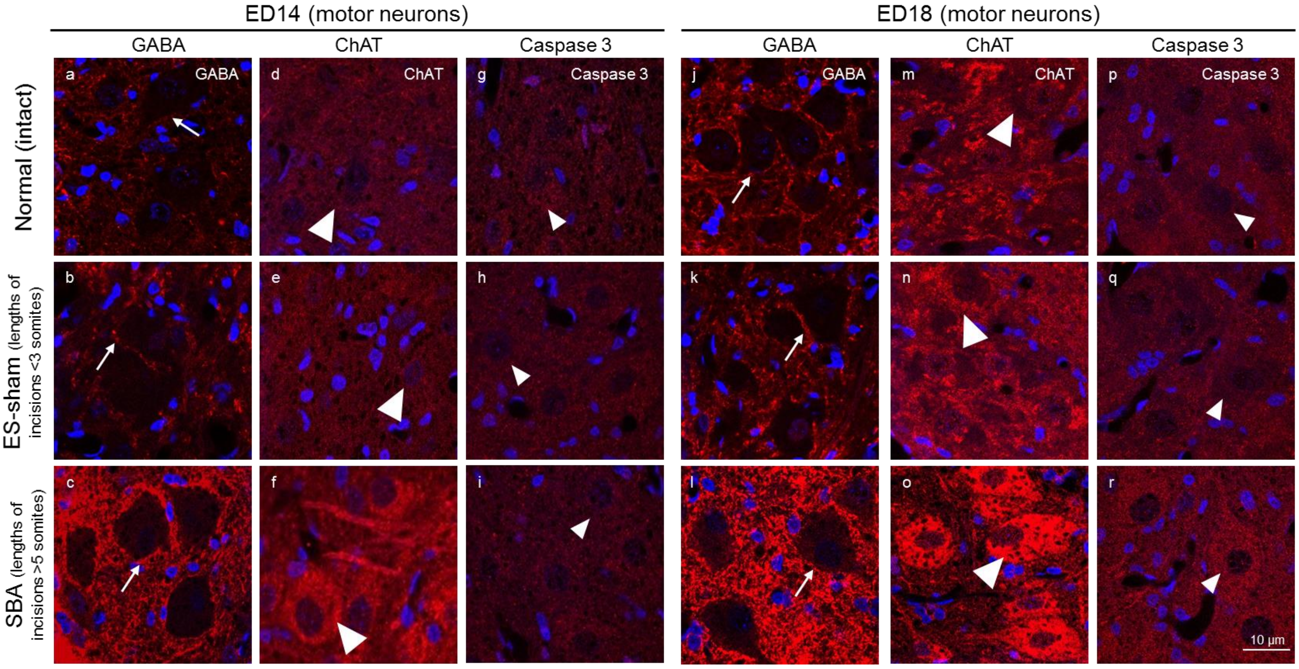

Supplementary Figure S4. Mechanical injuries at the embryonic stage did not influence GABAergic, cholinergic, or caspase 3 reactivity in the sham control chicks. Representative confocal images from the ventral horn at the location of open defect in the lumbar cord of SBA chicks and similar locations in the normal controls and ES-sham chicks at ED14 and ED18. Each panel represents the immunoreactivities of GABA (a–c and j–l; red, arrows) and DAPI (blue) or choline acetyltransferase (ChAT; d–f, and m–o; red, large arrowheads) and DAPI (blue) or caspase 3 (g–i and p–r; red, small arrowheads) and DAPI (blue). The sham injury, induced by early embryonic neural tube incision, had little or no influence on GABAergic, cholinergic, and caspase 3 activities in the SBA chicks.

Disease Models & Mechanisms • Supplementary information
